# Preparing effective medical illustrations for publication (Part 1): pixel-based image acquisition

**DOI:** 10.2349/biij.4.1.e11

**Published:** 2008-01-01

**Authors:** SC Wang

**Affiliations:** Department of Diagnostic Radiology, Yong Loo Lin School of Medicine, National University of Singapore, Singapore.

## INTRODUCTION

The explosion in medical literature over the last 20 years has seen a huge increase in the number of articles and books written, as well as an increase in the number of journals being published, often in increasingly narrow areas of research and educational focus. The net result has been an increase in the amount of literature that doctors have to go through in order to have some semblance of keeping up with the rapid pace of change and expansion in medical knowledge.

Even though the widespread availability of online tools such as PubMed, the Cochrane Database and even tools such as Google Scholar has made searching for publications and their key information easier than ever before, for radiologists at least, the publication of appropriately high quality medical images and illustrations remains of crucial importance for our highly visually oriented discipline.

For many busy radiologists reading hardcopy literature (despite online access and PDF soft copy versions, printed matter remains more portable, convenient and easier to read for many people than electronic versions of the same documents), there is a commonly adopted strategy for dealing with the very large and increasing body of published imaging literature:

- First skim the titles
- Then look at the pictures and illustrations
- Maybe read the abstract if these look interesting
- Occasionally, read the paper or chapter

Even the most erudite articles with the greatest scientific impact lack some force on the page without highly effective, well-designed and clear illustrations and images. In short, images help to “sell” the message of a radiology article or chapter, help to distill key points into graphical form, and help to ensure that the piece is actually read.

In the last decade, digital acquisition, storage and transmission of medical images has become widespread and pervasive. The time-honoured tradition of photographing the hardcopy film with a film camera to produce glossy prints for publication has almost disappeared as publishers have adopted totally digital workflows, and as radiologists have obtained increasing access to digital acquisition methods from DICOM images to film scanners and digital cameras. However, two things remain unchanged in this digital image era: a) the original source image has to be of good quality, and b) the image must still be optimised for the intended publication. Today publication can range from a low resolution web-page image to a huge mural-sized poster, and the images should be modified prior to export to a suitable standard image file format to ensure that the final reproduction is of suitable quality.

This article is the first of a two-part series which highlights and describes important approaches to producing high quality medical illustrations for publication, which has differing requirements to electronic, web-based or computer presentations. This part deals with the image sources available to radiologists, and how we should use them to capture images at an optimal quality suitable for publication, whatever the eventual medium. The second part will deal with software manipulation of such images and the use of dedicated illustration packages to create medical diagrams, annotations and charts.

## PREPARING FOR PUBLICATION

In general, hardcopy publication has significant constraints in length and the number and type of illustrations that can be included. This forces author(s) to be highly selective and careful in their choice of images that convey the key points in the most economical and effective manner.

Preparing for publication then depends on the type of piece that is to be authored. In general, case reports are the shortest publications and are limited to only a few illustrations pertinent to the specific case being reported. Subject or technique reviews, book chapters and pictorial essays need many more illustrations, and the emphasis is on breadth, range and quality of images, with often fairly lengthy captions. Original articles demand mainly well-organised tabulated data and charts, with usually fewer specific images and illustrations that highlight key points.

In general terms, all hard copy publications have usually very explicit, clearly defined written guidelines for authors. These are crucial and should be carefully read *before* embarking on creation of the graphics, since the ultimate output must be designed for the likely printed size, resolution and even potentially paper of the final product. So find the instructions, read them and then follow the guidelines when creating the images. Standardising on specific fixed image sizes and resolutions for publication greatly simplifies the eventual publication.

A medical image or set of images often need alphanumeric identification and appropriate arrows or other symbols highlighting specific features, with an appropriate legend. A caption is usually required in order to ensure the reader can quickly grasp what the images are intended to depict.

## PIXEL-BASED ILLUSTRATIONS

Today, all illustrations are digital in nature, or become so in the course of publication preparation, simply because publishing technology is now universally digital, with all journals now laid out using computers and dedicated professional software tools.

Pixel-based graphics are the mainstay of radiology; virtually all medical images are pixel-based. These also include photographs and scans of hardcopy images. The key aspect of these images is that they are resolution-dependent, and cannot be infinitely enlarged or shrunk without a sometimes major loss of quality. The key information all pixel-based images possess are listed in Table 1 .

**Table 1:** Pixel-based images and pixel

**Property**	**Units**	**Description**	**Example**
Total pixel count	Pixels	Individual picture elements, typically square, typically with brightness values 0-255	5 megapixels (i.e., 5 x 1024 x 1024 = 5,242,880 pixels)
Pixel dimensions	Pixels	Linear number of pixels for the rectangular image sides	2048 x 1920 pixels
Pixel density or resolution	Pixels per inch (ppi) or per cm (ppc)	Number of pixels along the side of a linear dimension (thus squared for the area of the same dimension)	72 ppi – typical for web and onscreen 200 ppi – typical for printed matter
Linear size	Inches or centimeters	Final length of each side for the image; dictated by the pixel dimensions and pixel resolution	7.5 x 10 cm 3 x 4 inches
Lossless formats	NA	Images preserving all the quality of the original. May be “losslessly compressed” without reducing quality (typically 2:1 compression). TIFF and PSD formats permit preservation of layer information, and are universally supported by professional publishing software	Photoshop (PSD) Tagged Image File Format (TIFF) Portable Network Graphics (PNG)
Native capture formats	NA	Images preserving all the quality of the original, but requiring significant editing (e.g., adjustment of window and level, resolution, colour mode etc) before being ready for print or screen publication	RAW (native capture) format, specific to camera manufacturer) DNG (Adobe digital negative, a “universal” RAW format) DICOM original files
Lossy formats	NA	Images which deliberately discard data to obtain much smaller file sizes to facilitate both storage and speed of distribution. GIF is very poor for continuous-tone images such as radiographs. High levels of JPEG compression cause marked loss of quality.	Joint Photographic Experts Group (JPEG) Graphics Interchange Format (GIF)

It should be clear from Table 1  that the linear size, very relevant to final printed output, is dependent on both the pixel resolution and the pixel dimensions of the image.

Vector images (drawings and charts) will be dealt with in the second part of this article.

### Image Resolution

For print and presentation applications, images are limited in *gamut*, or range of colours and shades of grey reproducible. Typically, only a small fraction of the 24-bit colour (16 million colours) and 8-bit grey-scale (256 shades of grey) range can be reproduced on paper, because of limitations in paper and ink technology. Furthermore, all grey-scale and colour printing involves a process called *line-screening*, where any given pixel’s shade of grey is reproduced by a pattern of small dots of varying size and ink density (see Fig. 1). This is because for routine applications, grey-scale printing is performed only with black ink, and colour printing with only 4 inks, the *CMYK* (*C*yan, *M*agenta, *Y*ellow and blac*K*) system. Shades of grey and colours have to be simulated by a variable dot density/size/colour pattern. There are sophisticated print methods using *stochastic screening* and other technologies to eliminate screening altogether, and some very high end printing presses use 6 or more colours in the process, but these are limited to niche publications and are not relevant to Radiology.

The line-screen resolution is invariably lower than the pixel resolution. Line-screen resolution is most commonly expressed in *lines per inch (lpi).* As a rule of thumb, an image’s pixel resolution should be 150-200% that of the line-screen resolution. Although higher resolutions can be used, this leads to much larger file sizes, longer image transmission times and printing times without any improvement in final quality.

Common line screen resolutions used for print applications and their corresponding pixel resolutions required are listed in Table 2.

**Table 2:** List of print/output applications, the image resolution required and its dependence on the publication line screen resolution.

**Output Application**	**Screen Resolution (lpi)**	**Image Resolution (dpi)**	Typical linear* pixel size for publication
Computer screen / web	NA	72 – 96	400 – 800
Newspaper	75	113 – 150	800 – 1200
Book chapter / journal	100 – 125	150 – 250	1000 – 2000
Glossy magazine / High-end printing	144 – 150	216 – 300	2000 – 4000

* *Linear pixel size* refers to the dimensions of the bounding rectangle of an image. A square image for book publication is typically 1000 x 1000 pixels in size.

### Native vs. Interpolated Resolution

We are so used to seeing medical and other images displayed on computer screens at varying resolutions that we do not appreciate that for the most part these are not displayed at the original resolution of the image, but rather are *interpolated*. This means that the display software artificially increases or decreases the number of pixels displayed to match the resolution and size of the screen.

Medical images range in native pixel size from 256 x 256 (65,536 or 64K pixels) for some CT, MRI and Ultrasound, up to 5 megapixels (MP) for computed radiography (CR) and 10 MP for digital mammography. If the native medical images were used directly for printing, a high end print publication could only reproduce a 256^2^ image at about 1 inch square and a 512^2^ image at 2 inches square, which is too small for normal use. Correspondingly, a digital mammogram or CR file is far too large for routine use and if unadjusted would lead to excessively large document and printing files. Furthermore, most DICOM medical images are typically 10-bit or 12-bits per channel for greyscale (i.e., there are thousands or millions of shades of grey), and computer displays and printed publications cannot reproduce more than 8-bit greyscale (256 shades of grey) routinely. All DICOM images must therefore be manipulated and adjusted prior to export into formats suitable for online or print publication.

There are a number of ways to obtain images for publication from medical imaging systems. Until the advent of the digital era, each medical image intended for publication underwent a lengthy and time-consuming multi-step process:

- The image was printed to hardcopy film, ideally without the patient name and distracting annotation. This was sometimes done as a separate print from the normal hardcopy for interpretation and storage.
- The image was photographed with a 35mm camera with appropriate cropping and scaling to fit the frame, and exposure adjusted to highlight specific features (especially for x-ray images)
- The negative was processed and printed to glossy paper prints at the size stipulated by the publisher. In order to capture the specific greyscale information of the key finding, this step was often repeated to alter the greyscale emphasis.
- The prints were labelled and any arrows, text or other overlay annotation added using sticky transfer lettering (e.g., Letraset).
- Once accepted, subsequently each image was rephotographed by the publisher and converted to a line screen image for printing.

It can be appreciated that these steps effectively reinterpolated the image size. A small image could be effectively scaled up optically in this manner, and a large one scaled down. This process meant the final image was 3 to 4 generations removed from the original, leading to a major loss of quality unless care was taken at each step.

Today this process is universally performed using software tools and all publishers have a completely digital workflow, ideally without modification to the submitted image. The commonest methods used are listed in Table 3.

**Table 3:** Image capture/export methods and their strengths and weaknesses.

**Source**	**Advantages**	**Disadvantages**
Photograph film image	• High quality assured if optimised imaging chain available • Not limited by fundamental image resolution but by print size and pixel interpolation	• Must print to film first (subject to artefacts) • Significant grey-scale range always lost • Minimal ability to adjust film image contrast etc.
Scan film image (dedicated film scanner required)	• High quality, high bit depth possible (e.g., 16-bit)	• Must print to film first (subject to artefacts) • Requires specialised film scanners (very expensive) • Some grey-scale range lost
Direct export of images from PACS or DICOM CD	• No intermediate print step • Avoid film artefacts (printer miscalibration, dust, scratches, fingerprints etc.) • Full high bit depth (e.g., 16-bit grey-scale) preserved	• Image sizes fixed by imaging system; may be too large or too small for print • Prints cannot manage more than 8-bit data for grey-scale; requires windowing adjustments first
DICOM browser image export	• No intermediate print step • No film artefacts • Allows accurate preview of image size, contrast and brightness prior to export	• Must have appropriate software and hardware platforms • Limited by computer screen size; cannot be precise size • Usually tedious, manual one-at-a-time export process

For most radiologists the best options are either a) photographing an already printed hardcopy image, or b) exporting an image correctly scaled and windowed to his/her liking from a DICOM image viewing software platform, either standalone or web-based through an image web server. Both of these methods produce typical computer file formats such as JPEG, TIFF etc, and are much faster and more convenient than scanning the film or attempting formal DICOM image export and subsequent conversion. In the case of photography, today this means using a digital camera, and for DICOM conversion and export, specialised DICOM viewing software (much of it freely available on the internet) that allows for such image conversion.

### Digital Cameras

The digital camera has become ubiquitous and relatively inexpensive, and is rapidly replacing the film camera for virtually all applications. It has several advantages:

- Fast, flexible, lightweight, portable
- Almost free and unlimited image capture
- Instant feedback on exposure and overall image appearance
- Easy input, storage, transmission and management
- Photograph any printed image
- Routinely high resolution pixel matrix (5 megapixels or higher)

However, digital cameras are not created equal, and the vast majority of available cameras are designed to photograph pictures for computer screen and snapshot printing output, with only limited control over image exposure and minimal control over in-camera processing. As such they are usually limited to 8 bits per channel, have limited or no means of capturing directly in grey-scale, and have problems with variable levels of image noise and lens-induced image distortion.

Today, digital cameras range in pixel count from at least 3 MP to 10 MP for common consumer applications. Paradoxically, as the above discussion shows, a typical printed image 10 cm (4 inches) square for a 150 lpi screen needs 300 x 300 x 4 (360,000) pixels, or far less than 1 megapixel. Thus, ANY modern digital camera available far exceeds the pixel resolution needed for good quality radiology image reproduction in print.

However, the demands of capturing optimal radiology images, especially from the extremely wide density range of x-rays and mammograms, require sophisticated controls within the camera. Although professional equipment is ideal, it is often very expensive. Today, the digital camera class known as “semiprofessional” or “advanced amateur” usually has the appropriate features needed for photographing radiology images with suitable image quality. These include:

- Very high quality lens, with macro photography option and minimal distortion
- Must be able to focus very close to the object (<6 cm minimum distance)
- Complete manual control of exposure
- Low noise sensor and/or large size digital sensor
- Ability to capture in RAW mode

In most cases this list means a digital single lens reflex (D-SLR) camera. However, a few high end “all-in-one” cameras do have such capabilities built in and can take very acceptable images, although in general these have more image noise than those from D-SLR’s. Finally, it should be noted that no available digital camera is able to capture the full greyscale range of a properly exposed x-ray or mammogram; optimal exposure requires the user knows exactly what he or she wishes to show on the final image.

### Using a Digital Camera

For photography of images printed to film, the minimum setup for consistent quality is a good evenly illuminated lightbox in a darkened room, some shutters or dark sheets of card or film to cut out excessively bright borders and a tripod. Ideal but much more cumbersome is a dedicated copy stand, with a flat horizontal lightbox and the camera mounted on a vertical arm.

Framing the image is important to save time and post-processing. Although it is tempting to attempt the cleanup work in Adobe Photoshop or similar pixel editing software, this is time-consuming and cannot correct for many problems. As much as possible, the best possible image should be obtained “in camera”. The image should be centred and square to the camera; i.e., the plane of the film should be parallel to the plane of the camera detector; otherwise the image will suffer from geometric distortion and will require software correction. Also, the camera should be positioned so that the desired image fills as much of the frame as possible with minimal extension into adjacent images or dead space. The exception to this is if distortion cannot be easily avoided by better positioning; software distortion correction always requires some “dead space” around the image.

If using a D-SLR with a dedicated macro lens, there will be no significant lens distortion — in short, straight lines will be straight throughout the entire image. However, most integrated digital cameras have zoom lenses that invariably show some *barrel distortion* (see Fig. 2) at the widest angle settings where the focussing distance is closest; this is manifest by outward curvature of straight lines near the edge of the image. Although this distortion can be corrected with appropriate software, this is too specialised and tedious for the general user, and is best avoided or minimised at the capture stage.

Most lenses have minimal barrel distortion at mid-zoom or telephoto settings, but the closest focussing distance is much greater and the maximum magnification is actually reduced. For most cameras, a mid-zoom range setting produces acceptable results, with still-useful close focussing and minimal distortion.

A tripod is generally mandatory because the exposure times for most cameras using a low ISO rating (50-100) to minimise image noise is in the range of 0.5 to 1/15 second; handshake is reliably eliminated only at exposures of 1/125 second or higher, so for most people the tripod is essential for consistent image quality. D-SLR cameras typically have very low image noise with high ISO ratings, which would allow for handheld exposures. It is possible to reduce, but not entirely eliminate such image noise using software tools (Fig. 3).

However, one of the major reasons for using a mechanical support is that it is much easier to consistently frame an exposure, especially when using a copy stand.

Obtaining the correct exposure can be difficult, especially for x-rays. The use of center-weighted or an averaged automatic exposure setting on the camera usually results in the correct exposure for most images. However, such averaged exposures can be problematic for mostly dark images (e.g., diffusion-weighted MRI with a single small bright lesion) or mostly bright images (e.g., narrow lung windows on HRCT of the chest). This is because the majority of the image is not grey; all automatic exposure systems are calibrated to ensure the midrange image intensity is forced towards the centre of the illumination scale (i.e., grey). So for very dark images the user should force the camera to deliberately under-expose by as much as –2 or –3 EV, and conversely over-expose bright images by as much as +2 or +3 EV. These steps will ensure that blacks appear black and white appears white on the final image.

For x-rays and mammograms, the density range is usually greater than the digital camera can capture. This means that exposure has to be optimised to show the feature of specific interest at its best, which may mean the overall image may be over- or under-exposed, and that spot metering or even manual exposure will be needed to ensure the appropriate finding is clearly visible. Sometimes, image exposure bracketing (3 or more exposures varying by ± 1 EV) is required to ensure accurate capture of the desired greyscale.

More recently, many cameras have been equipped with RAW image capture capability. This is standard on all available single-lens reflex digital cameras, but is only available on a limited range of high end “semi-professional” all-in-one cameras. The RAW file represents the original unmodified digital data captured by the camera sensor, and offers a major advantage over the typical JPEG or TIFF images produced by most cameras, in that no in-camera postprocessing is performed and often less image noise and more image detail, especially at the extremes of shadow and bright areas, can be retrieved by dedicated computer-based RAW image processing software. Applications such as Adobe Photoshop are now supplied with such processing capability as standard, and camera vendors routinely supply dedicated RAW processing software packages with their RAW-capable cameras.

Unfortunately, there is no industry standard method of creating a RAW image file format, and every vendor takes a different approach, sometimes significantly changing the format for different cameras in their own product lineup. It typically takes other software vendors some weeks, even months, to update their software to handle the latest file formats as new cameras are released. The end-user thus may be restricted in their choice of post-processing software. Adobe has created the closest thing to a common format, the DNG file, an intermediate format that almost all RAW image formats can be converted to using Adobe’s free DNG conversion software. However, this remains a somewhat unsatisfactory solution, requiring an extra step in image conversion in the workflow from camera to final image.

In addition, specialised processing software has been developed to create High Dynamic Range (HDR) images from 3, 5 or even more different wide-ranging exposures of a single subject. While this has been mostly confined to creating spectacular high resolution colour images of geography, scenery and paintings, it is ideally suited to the creation of exceptional quality capture of static medical images with a very wide dynamic range, such as x-rays and mammograms. The production of these images is however still tedious and somewhat experimental, and is neither within the scope of the average user or necessary for the vast majority of medical images.

While pressing the shutter button directly is simple enough, once a tripod has been set up and the image framed correctly, use of the self-timer system is ideal; most have the option for a 2-second delay, which is enough time for any vibration to settle before exposure.

The image resolution captured, as noted above, is generally far greater than needed for publication. Nevertheless, the maximum resolution, best quality images possible should be captured at all times. In-camera reduction of resolution to say, 2 megapixels, will result in considerable image data being discarded with potentially serious loss of quality. It is much safer and more controllable to capture at the maximum resolution possible and then adjust the image size using computer software afterwards.

Ultimately, wherever the highest quality is needed, the RAW format should be used if the camera supports it and the user is willing and able to use this less convenient approach.

### Exporting Images

Regardless of the image format at acquisition, whether from a DICOM image, film scanner or digital camera, page layout applications used by professional publishers require one of two standard formats in most cases: lossless Tagged Image File Format (TIFF) or high-quality, minimal lossy compression Joint Photographic Expert Group (JPEG) images. All pixel-based editing or capture applications will allow export or import of either of these two formats.

## CONCLUSIONS

The old computer adage, “garbage in, garbage out” applies very well to medical image capture for publication. We must always start with the best quality image possible; for many radiologists today this is often the original DICOM image itself, exported directly from the scanner or PACS system, and exported to JPEG or TIFF with the appropriate cropping, zoom and window-level settings to optimise the key feature of the image. The next best solution is a high quality print to film, which is then captured digitally either by a dedicated film scanner or carefully performed digital photography as described above. Older processes, such as photography of a paper print or the scanner display itself, are no longer considered acceptable.

There are many more steps required to ensure that a captured image becomes optimised for printing or publication; today these are universally post-acquisition manipulations in software applications on a computer, and are covered in Part 2 of this article.

